# Hereditary angioedema with normal C1 inhibitor associated with carboxypeptidase N deficiency

**DOI:** 10.1016/j.jacig.2024.100223

**Published:** 2024-02-01

**Authors:** Denis Vincent, Faidra Parsopoulou, Ludovic Martin, Christine Gaboriaud, Jacques Demongeot, Gedeon Loules, Sascha Fischer, Sven Cichon, Anastasios E. Germenis, Arije Ghannam, Christian Drouet

**Affiliations:** a Allergy and Internal Medicine Unit, University Hospital, Nîmes, France; b Centre de compétence, Centre de Référence des Angioedèmes (CREAK), Nîmes; c KininX SAS, Grenoble, France; d CeMIA SA, Larissa, Greece; e Dermatology Department, University Hospital, Angers, France; f Centre de Référence des Maladies Rares de la peau et des muqueuses d’origine génétique-Nord (MAGEC), filière FIMARAD, CHU Angers, Angers, France; g Université Grenoble Alpes, CEA, CNRS, IBS, Grenoble, France; h Université Grenoble Alpes, CHU Grenoble Alpes, Grenoble, France; i Human Genomics Research Group, Department of Biomedicine, University of Basel, Basel, Switzerland; j Institute of Medical Genetics and Pathology, University Hospital Basel, Basel, Switzerland; k Department of Immunology and Histocompatibility, University of Thessaly, Larissa, Greece; l Institut Cochin, INSERM, CNRS, Université Paris Cité, 75679, Paris, France

**Keywords:** Urticaria, angioedema, hereditary carboxypeptidase N deficiency, *CPN1* gene

## Abstract

**Background:**

Hereditary angioedema (HAE) is a potentially life-threatening disorder characterized by recurrent episodes of subcutaneous or submucosal swelling. HAE with normal C1 inhibitor (HAE-nC1-INH) is an underdiagnosed condition. Although the association with genetic variants has been identified for some families, the genetic causes in many patients with HAE-nC1-INH remain unknown. The role of genes associated with bradykinin catabolism is not fully understood.

**Objective:**

We sought to investigate the biological parameters and the genes related to kallikrein-kinin system in families with a clinical phenotype of HAE-nC1-INH and presenting with a carboxypeptidase N (CPN) deficiency.

**Methods:**

This study includes 4 families presenting with HAE-nC1-INH and CPN deficiency. Patients’ clinical records were examined, biological parameters of kallikrein-kinin system were measured, and genetics was analyzed by next-generation sequencing and Sanger sequencing. Predictive algorithms (Human Splicing Finder, Sorting Intolerant From Tolerant, Polymorphism Phenotyping v2, MutationTaster, and ClinPred) were used to classify variants as affecting splicing, as benign to deleterious, or as disease-causing.

**Results:**

Patients presented with angioedema and urticaria, mainly on face/lips, but also with abdominal pain or laryngeal symptoms. Affected patients displayed low CPN activity—30% to 50% of median value in plasma. We identified 3 variants of the *CPN1* gene encoding the catalytic 55-kDa subunit of CPN: c.533G>A, c.582A>G, and c.734C>T. CPN deficiency associated with genetic variants segregated with HAE-nC1-INH symptoms in affected family members.

**Conclusions:**

*CPN1* gene variants are associated with CPN deficiency and HAE-nC1-INH symptoms in 4 unrelated families. Genetic CPN deficiency may contribute to bradykinin and anaphylatoxin accumulation, with synergistic effects in angioedema and urticarial symptoms.

Hereditary angioedema (HAE) is a potential life-threatening disorder. It is characterized by recurrent attacks of subcutaneous or submucosal swelling. It can be disabling, and it affects various areas of the body including the face, the upper and lower limbs, tongue, uvula, lips, intestine, and larynx.^1^ The abdominal attacks are painful and are often accompanied by nausea, vomiting, and diarrhea.^1^ Edema may become life-threatening if the upper respiratory airways are involved. Disease diagnosis can be challenging, and patients are operated for unnecessary laparotomy in 27% of cases.^2^

HAE has been classified into different types: HAE with C1 inhibitor deficiency (HAE-C1-INH) resulting from variants in the *SERPING1* gene encoding C1-INH and HAE with normal C1-INH (HAE-nC1-INH), which includes variants in *F12*,^3^^,^^4^ *PLG*,^5^ *ANGPT1*,^6^ *MYOF*,^7^ *KNG1*,^8^ or *HS3ST6*.^9^ Although the association with genetic variant(s) has been identified for some families, the genetic causes in many patients with HAE-nC1-INH are unknown. The role of genes associated with bradykinin catabolism in the etiology of HAE is not fully understood.

Carboxypeptidase N (CPN, *alias* kininase I) is a zinc-metallopeptidase that specifically cleaves C-terminal basic residues, Arg and Lys, from biologically active peptides and proteins.^10^, ^11^, ^12^ CPN has been shown to cleave and thereby regulate (1) kinins, for example, bradykinin and Lys-bradykinin, generating *des*Arg^9^-bradykinin and *des*Arg^10^-Lys-bradykinin, respectively,^13^^,^^14^ with subsequent transformation of kinin B_2_ receptor ligands into B_1_ receptor ligands; (2) anaphylatoxins,^15^ for example, C3a and C5a, with generation of inactivated *des*Arg-C3a and *des*Arg-C5a; and (3) the chemokine stromal-derived factor 1α.^16^ Thereby CPN exerts control over multiple mediators of inflammation, vascular permeability, chemoattraction, and leukocyte activation and trafficking. CPN is composed of a 55-kDa catalytic subunit, encoded by *CPN1*, and a noncatalytic regulatory subunit, encoded by *CPN2*. The CPN2 protein protects the 55-kDa catalytic subunit from glomerular filtration, degradation, or enzymatic inactivation.^17^ CPN is biosynthesized by the liver in an active form and is present in plasma at a concentration of 30 to 40 mg/L.^6^ This high CPN concentration has been implicated in supporting an essential protective role from chronic inflammatory conditions.^9^^,^^12^^,^^15^

As shown by Zhou et al,^18^ mice lacking carboxypeptidase B2 (CPB2), CPN, or both plasma carboxypeptidases have enhanced vascular permeability implicating CPN deficiency in angioedema. Consequently, deficiency in either CPB2 or CPN activity should be considered in unexplained cases of HAE. Two cases of partial CPN deficiency have been documented (Online Mendelian Inheritance in Man 603103) in patients presenting with angioedema or urticarial episodes. The first case was of a 65-year-old man with 21% of normal CPN activity and nC1-INH, who presented with an 11-year history of severe recurrent angioedema occurring almost 40 times a year. His medical history included allergy and asthma, with elevated histamine during attacks.^10^^,^^19^ Genetic analyses revealed combined *CPN1* variants, NM_001308.2:c.[173dup(;)533G>A;p.(Gly178Asp)], which were suggested to cause the clinical phenotype.^20^^,^^21^ The second case was of a female with CPN deficiency and iatrogenic angioedema 2 years after starting treatment with an angiotensin I–converting enzyme inhibitor (ACEi), although the background for this case was not fully described.^22^

In this article, we present 4 families with HAE-nC1-INH clinical phenotype and CPN deficiency. The biological parameters and selected genes related to kallikrein-kinin system (KKS) were investigated.

## Methods

### Study subjects

Four unrelated families were included in the European Program on Rare Diseases No1 (E-RARE-1) research program on HAE-nC1-INH. Probands and several family members were investigated. All individuals tested had nC1-INH and complement levels compared with healthy blood donors; a diagnosis of HAE-nC1-INH was issued for family probands. Healthy blood donor samples served as controls.

### Sampling procedures and laboratory methods

EDTA blood samples were used for genetic analyses. Citrated plasma samples were used to investigate bradykinin metabolism. Samples were collected outside the window of ACEi intake. Plasma samples were immediately frozen and kept at −80°C until analysis.

CPN activity was measured according to a protocol modified from Skidgel^23^ using a FurylAcroyloyl-Ala-Lys substrate (Sigma-Aldrich, Saint Quentin Fallavier, France) that does not cross-react with circulating CPB2. Aminopeptidase P (APP) activity was assessed as previously described^24^ using *Abz*-Lys-Phe-Arg-Ser-Ser-Lys-Gln-EDDnp (ProteoGenix, Schiltigheim, France). ACE activity was measured using the ACE Kinetic kit (Bühlmann Laboratories, Allschwil, Switzerland), and plasma kallikrein activity in line with our study methods was investigated using an H-D-Pro-Phe-Arg-*p*NA substrate (ProteoGenix).^25^

### Genotyping

Genomic DNA was isolated using MagNA Pure (Roche, Meylan, France). DNA samples from probands of families B and C were analyzed by next-generation sequencing (Ampliseq custom panel, Thermo Scientific, Waltham, Mass), as described^26^ (see Table E1 in this article’s Online Repository at www.jaci-global.org for the genes submitted to the analysis). Briefly, DNA libraries were constructed for each sample using Ion AmpliSeq Library Kit 2.0 (Thermo Scientific) and indexed with a unique adapter using the Ion Xpress barcode adapter kit (Thermo Scientific). Template preparation, enrichment, and chip loading were carried out on an Ion Chef system (Thermo Scientific). Sequencing was performed on S5XL on 520 and 530 chips, using the Ion 510, Ion 520, and Ion 530 Kit-Chef workflow. Primary data were analyzed using the Ion Reporter software (Thermo Scientific). Sanger sequencing of exon 3 (CPN1ex3_F: 5′-AGTATTCAATCTGAAACCTTCATTTTT-3′, CPN1ex3_R: 5′-AGATGGCTTAGCAGTCTTTCTG-3′) was used to confirm *CPN1* variants and to sequence DNA samples.

### Bioinformatics

*In silico* prediction tools specifically designed for mutation evaluation were used with a number of software packages: Human Splicing Finder (https://hsf.genomnis.com), Sorting Intolerant From Tolerant (SIFT; https://sift.bii.a-star.edu.sg/), Polymorphism Phenotyping v2 (https://genetics.bwh.harvard.edu/pph2/), MutationTaster (https://mutationtaster.org), and ClinPred (https://sites.google.com/site/clinpred/). To determine minor allele frequency, we used the Genome Aggregation Database v3.1.1 (gnomAD; gnomad.broadinstitute.org/). Clinical interpretation of genetic variants scored by the American College of Medical Genetics and Genomics (ACMG)/Association for Molecular Pathology (AMP) 2015 guideline has been performed using InterVar (https://wintervar.wglab.org/).^27^

### Postanalytical phase

We identified variant positions consistently with recommendations of the Human Genome Variation Society and used terminology of angioedema in accordance with the DANCE (definition, acronyms, nomenclature, and classification of angioedema) initiative. Variant pathogenicity criteria were determined according to ACMG,^27^ and variant pathogenicity was curated according to a recent international consensus on HAE genetics.^28^

### Statistical analysis

Individuals from 4 families were stratified by enzymatic activities of plasma kallikrein, CPN, APP, and ACE. Reference interval was based on measurements of healthy blood donors: 98 male (20-68 years) and 101 female (18-67 years) donors. The Kolmogorov-Smirnov statistical test of normality (*D*) provided a measurement of the divergence of sample distribution from the Gaussian distribution. When the *D* score is nearer to 0, the more likely the distribution is normal. *D* values calculated for kallikrein and CPN activity in controls ranged from 0.0376 to 0.1155, in line with data distribution not significantly deviating from a Gaussian distribution, making the Mann-Whitney test applicable in statistical analysis. The percentile distribution of the reference population has been developed for biological parameters, with position of median values for comparison of patients with healthy controls. The nonparametric Mann-Whitney *U* test (Prism 8, GraphPad, Boston, Mass) compared values of individual and healthy controls. A *P* value less than .05 (typically ≤.05) was statistically significant.

### Ethics

All procedures were performed in accordance with the principles of the Helsinki Declaration and French ethical policies governing the use of biological sample collections (Ministry of Health declaration no. 2008-634). Informed written consent for molecular genetics analysis was obtained from patients in the presence of the physician. Patients consented to participating in an investigation with biological assays. The institutional review board (IRB 5891) at CECIC Rhône- Alpes-Auvergne (Clermont-Ferrand, France) stated on August 23, 2021, that the processing methods and data management met requirements. All data were processed anonymously.

## Results

### Clinical phenotypes

Clinical observations for all cases are provided in Table I.

**Table I:** Clinical records

Family ID	Patient	Sex	Symptoms∗								
			Peripheral	Abdominal	Laryngeal	Macroglossia	Urticaria	Trigger	Age of onset	Delay diagnostic	Treatment prophylaxis
A	I.2	F	Yes	Yes	No	No	No	Unknown	40 y	1 y	Tranexamic acid
	II.1†	F	Yes	Yes	Yes	No	Yes	Pressure pruritus, triptorelin	41 y	2 y	Tranexamic acid, icatibant on demand
	II.2	M	Yes	Yes	Yes	No	Yes	Unknown	—	—	Tranexamic acid
	II.3	F	Yes	Yes	Yes	No	Yes	Unknown	—	—	None
	III.1	F	Yes	Yes	Yes	No	Yes	Unknown	—	—	None
B	I.1	M	No	No	No	No	Yes	Unknown	22 y	75 y	None
	I.2	F	Yes	Yes	No	No	Yes	None	27 y	70 y	None
	II.1†	F	Yes	Yes	Mild	No	Yes	Spontaneous and/or cold	30 y	12 y	Tranexamic acid, montelukast, icatibant on demand
	III.1	F	Yes	Yes	No	No	Yes	Unknown	25 y	1 y	Tranexamic acid, montelukast, icatibant on demand
	III.3	F	No	No	No	No	Yes	Unknown	17 y	1 y	None
	IV.1	M	No	Yes	No	No	Yes	Unknown	12 y	6 mo	None
C	II.2	F	Yes	Yes	No	No	Yes	Cold	47 y	6 mo	None
	III.1	F	No	Yes	No	No	Rare	Unknown	17 y	6 mo	None
	III.2†	F	—	Yes	No	No	Yes	Cold	15 y	6 mo	Tranexamic acid, icatibant on demand
D	I.1	M	No	Yes	No	No	Yes	Unknown	16 y	30 y	None
	I.2	F	No	No	No	No	Yes	Unknown	12 y	30 y	None
	II.1†	M	Yes	Yes	Yes	Rare	Chronic urticaria	Pressure, cold, fatigue	18 y	14 y	Tranexamic acid, montelukast, icatibant, or C1-INH concentrate on demand

*F*, Female; *M*, male.

∗ Urticarial lesions in CPN-deficient patients developed frequently, but not consistently, in association with angioedema attacks. An urticarial rash accompanied nearly 60% of symptomatic episodes of angioedema.

† Family proband.

In family A, the proband II.1 presented with recurrent urticaria and peripheral angioedema triggered for the first time after stimulation for *in vitro* fertilization because of endometriosis. Symptom relief was observed after taking 3 g/d tranexamic acid and on-demand icatibant. Her mother I.2 and her brother II.2 were also symptomatic for angioedema, with symptom relief after taking 3 g/d tranexamic acid.

In family B, the proband II.1 presented with recurrent urticaria and episodic peripheral angioedema, with symptoms starting when the proband was under oral contraception. H_1_ antihistamines, even at the highest dose (20 mg/d desloratadine), failed to relieve symptoms. Symptoms were relieved by 3 g/d tranexamic acid and 10 mg/d montelukast, and on-demand icatibant in case of severe episodes. The daughter III.1 presented with a similar phenotype to the mother, with symptoms starting when she was under antiandrogen treatment; she successfully responded to the same treatments as the mother. Four other family members—I.1, I.2, III.3, and IV.1—presented also with the same symptoms.

In family C, the proband III.2 presented with recurrent H_1_ antihistamine–resistant urticaria episodes and abdominal attacks. Symptom relief was observed after taking 3 g/d tranexamic acid and on-demand icatibant. Two other family members—II.2 and III.1—were also symptomatic.

In family D, the proband II.1 presented with cold urticaria and H_1_ antihistamine–resistant angioedema (up to 20 mg/d cetirizine). Symptoms were relieved by administration of 3 to 5 g/d tranexamic acid and 10 mg/d montelukast, plus on-demand icatibant. Both parents presented a moderate phenotype. The proband described fatigue and stress as triggers of angioedema attacks.

The effectiveness of icatibant on the relief of severe episodes in all 4 families suggests at least partial involvement of bradykinin in the clinical phenotype. All probands were born from nonconsanguineous parents.

### Biological characteristics of the patients

Antigenic C1-INH and function were in the normal range for all individuals. Plasma CPN activity was significantly below the reference interval for all symptomatic patients (Table II). Plasma CPN activity when measured during the attacks was equivalent to levels measured in the intercritical period. Both APP and ACE activities were in the normal ranges (Table II). Kinin catabolism enzyme activities were measured in all patient samples at various time points over the years of patient follow-up and remained unchanged throughout the study (not shown). Spontaneous kallikrein activity was in the normal range for all subjects. However, kallikrein activity in dextran sulfate–stimulated plasma samples from patients I.2, II.1, III.1, and IV.1 of family B were lower than the normal range, suggesting that these subjects have low proenzyme levels. Interestingly, proband II.1 in family B had the variant c.689T>A on *KLKB1* gene, which may have contributed to the lower levels of proenzyme in this family (Table II). An increased proportion of high-molecular-weight kininogen was cleaved in samples from proband II.1 in family B collected during angioedema attack compared with sample obtained during the intercritical period (not shown).

**Table II:** Biological data

			Plasma kallikrein (*V*_max_, nmol/min/mL)				
			Spontaneous kallikrein activity	Proenzyme activation	CPN (*V*_max_, nmol/min/mL)∗	APP (*V*_max_, nmol/min/mL)	ACE (IU)
Reference population, 5th-95th percentile interval (median)							
	Male (n = 98)		3.1-9.2 (5.8)	1830-2765 (2253)	70.9-105.9 (92.0)	0.25-3.08 (1.04)	43-95 (69)
	Female (n = 101)		3.2-10.6 (6.4)	1870-2985 (2351)	79.6-100.0 (90.7)	0.50-5.93 (1.59)	42-85 (62)
**Family ID**	**Patient**	**Sex**					
A	I.2	F			**65.1 ± 11.1** (n = 2)†		
	II.1‡	F	1.7	3237 ± 136 (n = 2); NS	**27.2 ± 0.9** (n = 3)§	2.68 ± 0.18 (n = 3)	42 ± 4 (n = 3)
	II.2	M			**46.4 ± 7.9** (n = 2)||		
	II.3	F			82.5 ± 6.8 (n = 2); NS		
	III.1	F			86.7 ± 3.8 (n = 3); NS		
B	I.1	M	9.1	1819 **±** 434 (n = 2)†	**60.4 ± 1.2** (n = 2)||		
	I.2	F	5.7	**1316 ± 65** (n = 2)¶	**61.8 ± 1.7** (n = 2)¶		
	II.1†	F	7.7	**1233 ± 48** (n = 2)¶	**42.6 ± 1.5** (n = 2)||	0.82	53
	III.1	F	3.2	**1156 ± 43** (n = 2)¶	**62.4 ± 4.8** (n = 2)¶	0.67	47 ± 7 (n = 2)
	III.3	F	10.6	1896 ± 15 (n = 2); NS	**59.7 ± 4.7 (**n = 2)||	1.87	37.5 ± 3.5 (n = 2)
	IV.1	M	2.7	**1185 ± 106** (n = 2)||	**50.7 ± 4.2** (n = 2)||	0.46	78
C	II.2	F	10.5	3313	**38.4 ± 7.6** (n = 2)||		38.5 ± 0.7 (n = 2)
	III.1	F			**58.7 ± 1.1** (n = 2)||		
	III.2†	F	10.1	2808	**33.4 ± 1.5** (n = 2)||	0.90	41 ± 1.4 (n = 2)
D	I.1	M	3.9	3010	**70.6 ± 2.6** (n = 2)†	1.56	43
	I.2	F	4.2	2554	**63.7 ± 0.9** (n = 2)†	5.07	63
	II.1†	M	8.4	2883	**54.6 ± 0.4** (n = 2)||	1.89	75

Enzymatic continuous variables in patient samples are presented as mean **±** SD. Reference intervals with medians generated in healthy donors are presented (95th percentile). Enzymatic activity outside the reference interval is highlighted in bold.

*F*, Female; *M*, male; *NS*, not significant.

∗ Lower threshold value 0.7 nmol/min/mL.

† When compared with reference interval, significance of data is shown: *P* ≤ .05 (Mann-Whitney *U* test).

‡ Family proband.

§ When compared with reference interval, significance of data is shown: *P* ≤ .0001 (Mann-Whitney *U* test).

|| When compared with reference interval, significance of data is shown: *P* ≤ .001 (Mann-Whitney *U* test).

¶ When compared with reference interval, significance of data is shown: ∗∗*P* ≤ .01 (Mann-Whitney *U* test).

### Genetic investigation

**Fig 1:**
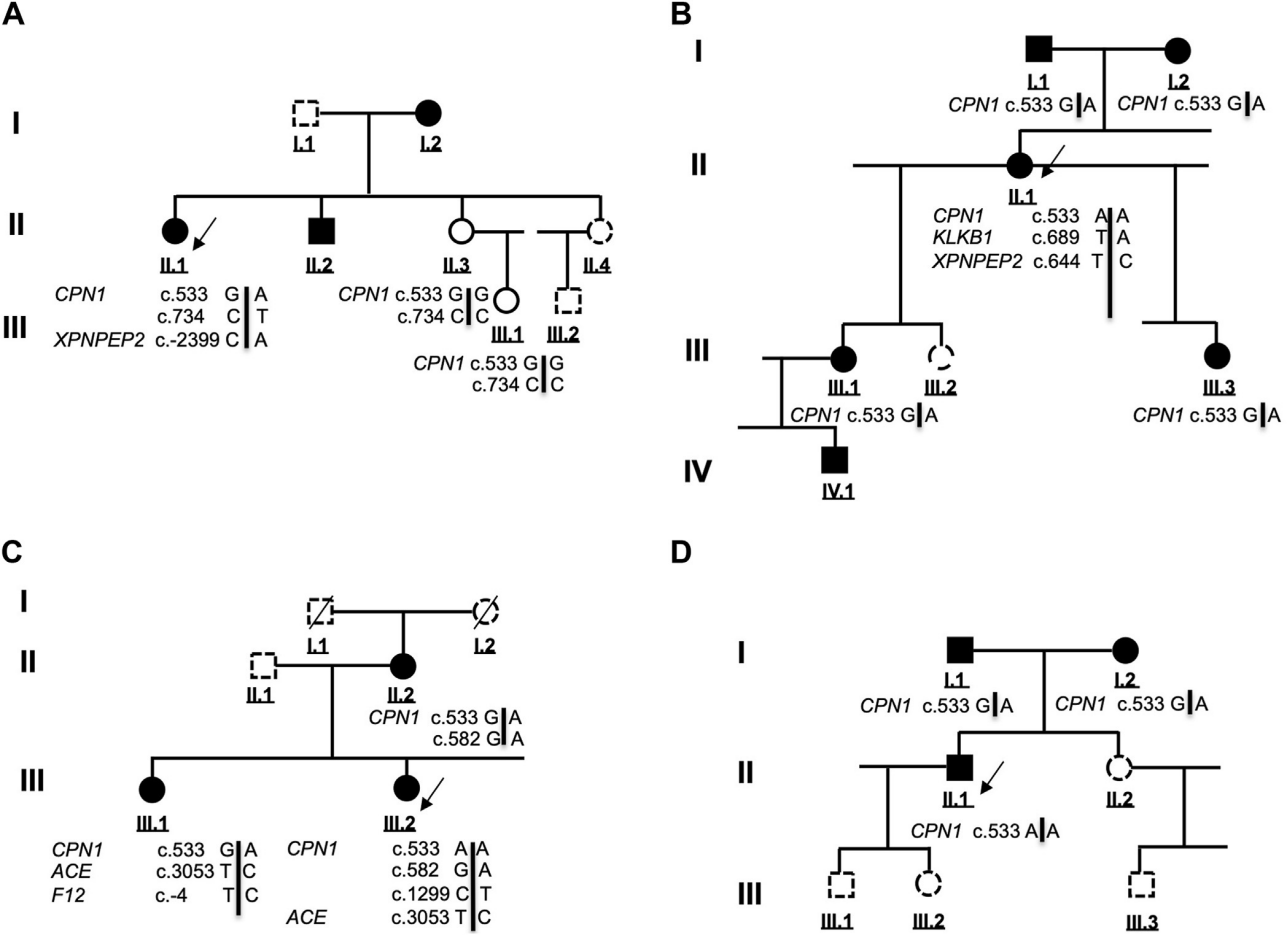
Pedigrees of families **A-D** presenting with a CPN deficiency. Results of next-generation sequencing and Sanger sequencing analyses. The *CPN1* variants c.533G/A, c.582G/A, c.734C>T, and c.1299C>T and additional variants cosegregating with clinical symptoms are presented. *Filled symbol*: individual affected by recurrent angioedema possibly associated with urticarial lesions; *empty symbol*: asymptomatic subject; *dashed symbol*: noninvestigated family member. *Arrows* indicate the probands of investigated families.

DNA samples from individuals in families B (II.1) and C (III.2 and III.1) were investigated by next-generation sequencing; material from all the other individuals was subjected to Sanger sequencing. Fig 1 shows the pedigrees and the allele distribution revealed by these analyses. Within the 4 families, *CPN1* variants associated with clinical symptoms and low CPN activity. Table III provides the genetic findings and associated bioinformatics data recorded for genetic variants.

**Table III:** Variants found in the 4 families presenting with CPN deficiency: Bioinformatic analysis

Gene	Encoded protein	Protein function	OMIM	Variant	MAF (gnomAD)		Prediction algorithms				
					World	European	HSF 3.0	SIFT	PolyPhen-2	MutationTaster	ClinPred
*CPN1*	Carboxypeptidase N, subunit 1	Kininase I	603103	c.533G>A p.(Gly178Asp) rs61751507	0.0034	0.0048		Deleterious (0.02)	Probably damaging (0.996)	Polymorphism (1.37 × 10^−7^)	Damaging (0.997)
				c.582A>G p.(Glu194=) rs190183597	0.000058	0.000061	Affects splicing				
				c.734C>T p.(Thr245Met) rs3710700915	0.000032	0.0000309		Deleterious (0.00)	Probably damaging (0.998)	Disease-causing (0.999)	Damaging (0.753)
				c.1299C>T p.(His433=) rs61733667	0.02721	0.03546					
	*Associated variants*										
*ACE*	Angiotensin I–converting enzyme	Kininase II	106180	c.3053T>C p.(Ile1018Thr) rs4976	0.00115	0.000037		Deleterious (0.00)	Probably damaging (0.999)	Disease-causing (0.999)	Tolerated (0.099)
*F12*	Factor XII, *alias* Hageman factor	KKS	610618	c.-4T>C rs1801020 common SNP	0.6522	0.348030					
*HRH1*	Histamine H_1_ receptor	Endothelial H_1_ histamine receptor	600167	c.42G>A p.(Met14Ile) rs79314450	0.0014	0.00053		Tolerated (0.058)	Possibly damaging (0.541)	Disease-causing (0.946)	Tolerated (0.024)
*KLKB1*	Plasma prekallikrein	KKS	229000	c.689T>A p.(Ile230Asn) rs142420360	0.000180	0.0003406		Deleterious (0.00)	Probably damaging (0.999)	Disease-causing (0.992)	Tolerated (0.315)
*MASP2*	Mannan-binding lectin associated protease 2	Complement convertase	613791	c.352C>T p.(Arg118Cys) rs147270785	0.00051	0.00064		Deleterious (0.02)	Benign (0.143)	Disease-causing (0.999)	Tolerated (0.173)
*MPO*	Myeloperoxidase	Neutrophil/mast cell granule enzyme	606989	c.752A>G p.(Met251Thr) rs56378716	0.01259	0.01317		Deleterious (0.00)	Benign (0.032)	Disease-causing (0.999)	Tolerated (0.088)
*SERPINC1*	Antithrombin III	Control of coagulation, KKS, and plasmin	107300	c.749C>T p.(Thr250Ile) rs144084678	0.00003	0.00005		Deleterious (0.00)	Probably damaging (0.993)	Disease-causing (1.00)	Damaging (0.841)
*SERPING1*	C1-INH	Control of KKS and complement	606860	c.1438G>A p.(Val480Met) rs4926	0.212	0.274		Tolerated (0.084)	Benign (0.034)	Polymorphism (0.999)	Tolerated (0.037)
*XPNPEP2*	Membrane X-prolyl aminopeptidase (APP)	Membrane kininase	300145	c.-2399C>A rs3788853	0.2261	0.2233					
				c.644C>T rs138365897	0.00236	0.00349		Deleterious (0.01)	Possibly damaging (0.871)	Disease-causing (0.995)	Tolerated (0.043)

The MAFs detected in the World and European populations are indicated according to gnomAD. The results from 4 prediction algorithms applied to missense substitutions are summarized: SIFT (with score), PolyPhen-2 (with probability score), and MutationTaster and ClinPred (with probability). The SIFT and PolyPhen-2 algorithms give scores ranging from 0 to 1. A mutation is predicted as “deleterious” by SIFT if its score is less than 0.05; otherwise it is predicted as “tolerated.” A mutation is predicted as “possibly damaging” by PolyPhen-2 if its score is greater than 0.15 and as “probably damaging” if it is greater than 0.85; otherwise it is predicted as “benign.” The MutationTaster algorithm indicates the probability of an alteration being a polymorphism or a disease-causing alteration. The scores range from 0 to 1, with a score of 1 indicating a high security of prediction. ClinPred incorporates machine-learning algorithms that use existing pathogenicity scores and benefits from normal population allele frequency. HSF 3.0 has been used as an algorithm for prediction of a synonymous variant.

*gnomAD*, Genome Aggregation Database v2.1.1; *HSF 3.0*, Human Splicing Finder system; *MAF*, minor allele frequency; *OMIM*, Online Mendelian Inheritance in Man; *PolyPhen-2*, Polymorphism Phenotyping v2; *SIFT*, Sorting Intolerant From Tolerant; *SNP*, single nucleotide polymorphism.

In family A, the proband II.1 was a female carrying compound heterozygous *CPN1* gene variants NM_001308.2:c.[533G>A];[734C>T]. The variants included a known variant c.533G>A;p.(Gly178Asp) and a new variant NM_001308.2:c.734C>T;p.(Thr245Met). Average frequencies of 3.4 × 10^−3^ and 3.2 × 10^−5^, respectively, were determined according to gnomAD, that is, 1.09 × 10^−7^ for the variant combination. The c.533G>A;p.(Gly178Asp) variant has been characterized as benign in ClinVar although it was suggested as pathogenic in the initial report^20^ wherein it was correlated with functional CPN deficiency. The p.(Gly178Asp) and p.(Thr245Met) variants are both located in signature motifs—PM14-Zn carboxypeptidase for Gly_178_ and Zn-binding signature for Thr_245_. These 2 residues are highly conserved residues across species and have corresponding positions in carboxypeptidase M,^29^ a glycosylphosphatidylinositol (GPI)-anchored carboxypeptidase expressed on endothelial cells.^12^^,^^21^^,^^30^ The 2 variants are predicted to be deleterious (SIFT), damaging (ClinPred), probably damaging (PolyPhen-2), and polymorphic for p.(Gly178Asp) or disease-causing for p.(Thr245Met) (MutationTaster) (Table III). The observations of both variations meet the ACMG criteria PS3, PS4, PM1, PM2, PP3, PP4, PP5, and BP6 specifically for p.(Gly178Asp), along with a pathogenic (recessive) characterization as evaluated by InterVar.

In family B, the proband II.1 was a female carrying homozygous variants NM_001308.2:c.[533G>A];[533G>A];p.(Gly178Asp), with a frequency of 1.15 × 10^−5^ for the homozygous combination; the same combination was detected in family D. The observations meet ACMG criteria PS3, PS4, PM1, PM2, PP3, PP4, PP5, and BP6, along with a pathogenic (recessive) characterization as evaluated by InterVar. An additional variant was found in this family: NM_000892.3:c.689T>A;p.(Ile230Asn) in the *KLKB1* gene, located in the Apple 3 domain of KLKB1 (prekallikrein), and predicted to be deleterious (SIFT), probably damaging (PolyPhen-2), disease-causing (MutationTaster), and tolerated (ClinPred). P.(Ile230Asn) may be putatively responsible for the recurrent low proenzyme content detected in individuals I.2, II.1 (proband), III.1, and IV.1 (Table II), recognized as a likely pathogenic (recessive) variant (frequency 1.8 × 10^−4^) in a prekallikrein-deficient individual^31^; it is not reported in ClinVar.

In family C, the female proband III.2 was homozygous for *CPN1* variant NM_001308.2:c.[533G>A];[533G>A];p.(Gly178Asp). Her sister III.1 was a compound heterozygous carrier of *CPN1* variants NM_001308.2:c.[533G>A];[c.582A>G]. Both individuals also carried a c.1299C>T;p.(His433=) variant, a single nucleotide polymorphism identified as rs61733667, not reported in ClinVar. The rare NM_001308.2:c.582A>G;p.(Glu194=) variant, with an average frequency of 5.8 × 10^−5^ (gnomAD), is predicted to affect splicing by activation of a cryptic exon acceptor site (Human Splicing Finder system 3.0); it is also unreported in ClinVar. The combination of both variants occurs at a frequency of 1.09 × 10^−7^. The observations for c.582A>G meet the ACMG criteria PS3, PS4, PM2, PM3, PP3, and PP4, along with a characterization as pathogenic (recessive) as evaluated by InterVar. Variant c.533G>A, a homozygous variant in combination with variant c.582G>A;p.(Glu194=), segregated with the clinical phenotype for patients II.2, III.1, and III.2. An additional variant was found in this family: NM_000505.3:c.-4T>C;rs1801020 polymorphism in the *F12* gene that has been recognized as a disease modifier in families with HAE-C1-INH^32^ and with HAE with a gain of function of factor XII (HAE-FXII).^33^

In family D, the male proband II.1 was found to be a homozygous carrier of NM_001308.2:c.[533G>A];[533G>A];p.(Gly178Asp)—the same combination as recorded in family B.

The clinical and genetic records from these 4 families, in line with the biological findings, are consistent with a hereditary CPN deficiency according to the following criteria:

1. Plasma CPN activity in proband samples significantly lower than 50% of the median value of healthy controls and below the lowest percentiles of a normal distribution of male and female controls (Table II);
2. *CPN1* variants segregating in individuals presenting a clinical history of urticaria and angioedema;
3. c.533G>A;p.(Gly178Asp) variant inherited in an autosomal-recessive pattern, suggested to be the disease-causing mutation when present in homozygous form—proband II.2 (family C) and proband II.1 (family D)—or in compound heterozygous form, for example, when combined with c.734C>T;p.(Thr245Met) for proband II.1, family A, or with c.582A>G;p.(Glu194=) for patient III.1, family C (Fig 1); the frequency of variant combinations is in agreement with the expected occurrence of HAE-nC1-INH;
4. *CPN1* alleles combined with pathogenic variants/gene modifiers in genes involved in kinin metabolism, for example, *KLKB1* and *F12* (Table III), with potentially partial contributions to chronic symptomatology.

## Discussion

This study presents 4 families with HAE-nC1-INH and CPN deficiency. Plasma CPN activity was significantly below the reference interval for all symptomatic patients with angioedema during critical and intercritical periods (Table II). In these families, CPN deficiency is associated with combinations of *CPN1* variants, with c.533G>A when present on both alleles or in combination with c.582A>G or c.734C>T. The variants were transmitted as an autosomal-recessive trait, and combinations of *CPN1* alleles cosegregated with angioedema clinical symptoms in patients. A high female-to-male ratio of CPN deficiency is in agreement with the observation of HAE-nC1-INH, including HAE-FXII.

Two major pathophysiological mechanisms of angioedema can be distinguished by the endotypes: (1) mast cell activation and (2) kinin dependency.^34^ Our findings are consistent with previous reports of CPN deficiency as described in 2 patients presenting with angioedema and/or urticarial episodes.^19^^,^^22^ Although symptoms of erythema marginatum, with susceptibility to icatibant, are not uncommon in patients with HAE-C1-INH during prodromal symptoms,^35^, ^36^, ^37^ the urticarial episodes are very rare.^38^ Besides, the issue of angioedema- or urticaria-specific triggers of attacks for CPN-deficient patients is difficult to address. Frequently, these patients presented with urticaria concomitantly with angioedema symptoms.

It could be hypothesized that as kallikrein promotes C5 and C3 cleavage and subsequent generation of anaphylatoxins,^39^^,^^40^ triggers of KKS activation could generate both anaphylatoxin and kinin ligands with subsequent synergistic effects on the clinical phenotype.

A circulating inhibitor as responsible for decreased CPN activity has been excluded after dilution experiments of patient plasma in sample from healthy donors (not shown). Rather, CPN deficiency was linked to a defective enzymatic activity or decreased level of the protein,^19^ associated with 3 *CPN1* variants that have been shown to segregate with the clinical phenotype and a low CPN activity.

CPN mediates approximately 10% of the catabolism of bradykinin in plasma, and CPN deficiency could contribute to the accumulation of bradykinin.^41^ The observation that the affected probands from the 4 families obtain symptom relief after administration of icatibant, a B_2_ receptor antagonist, implicates bradykinin in angioedema symptoms.

**Fig 2:**
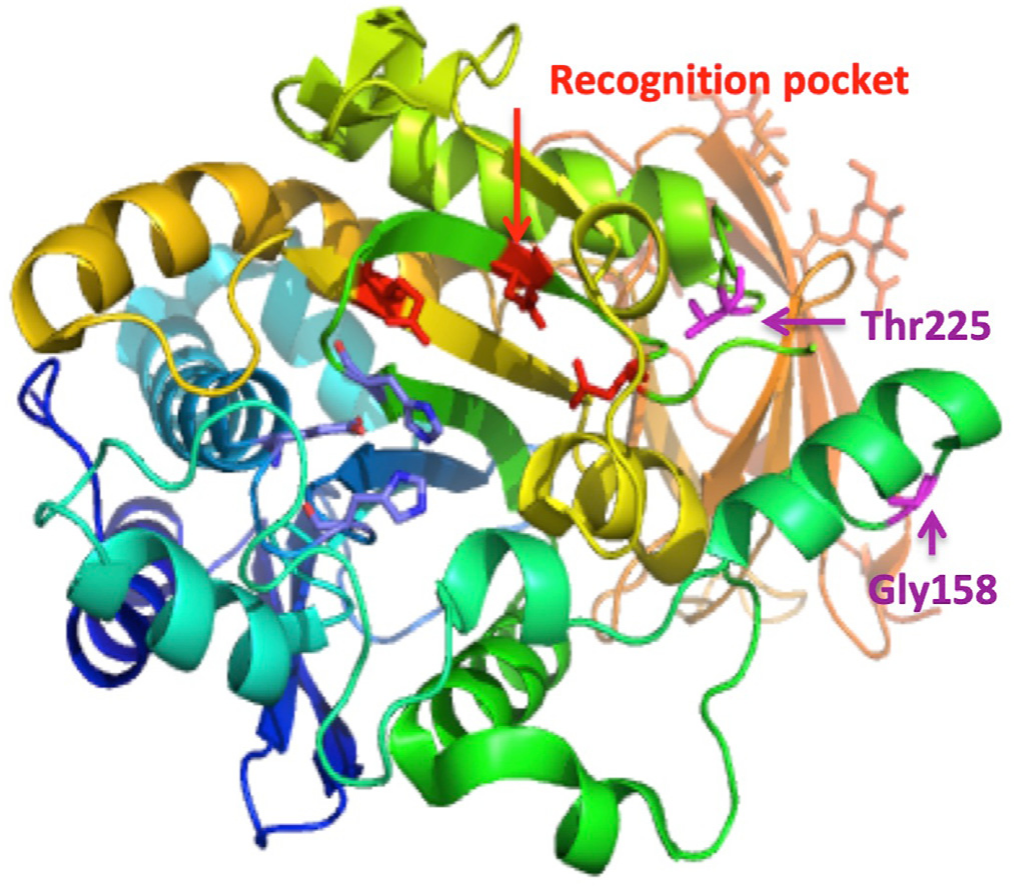
Expanded view of the CPN 3-dimensional structure (PDB #2NSM) showing the position of CPN1 variants described in the study. The catalytic triad is labeled *red*, and the catalytic Zn^2+^-binding site is indicated in *blue*. Residues lining the Arg/Lys binding pocket are labeled *red*, and the Zn^2+^-binding and catalytic residues are colored *blue*. Residues are numbered according to positions in the mature protein.

CPN deficiency was initially attributed to a combination of *CPN1* variants, c.[173dup](;)[533G>A].^20^ The 3 *CPN1* variants carried by individuals in these families were characterized with frequencies ranging from 3.2 × 10^−5^ to 3.4 × 10^−3^ (Table III) and variant combination frequencies that are compatible with a rare disease. Missense variants on both alleles at 1 of the 2 positions highlighted in Fig 2, built from PDB #2NSM,^42^ reduce—but not completely abrogate—catalytic activity (see Table II). Both Gly_178_ and Thr_245_ are strictly conserved throughout evolution^29^ and are therefore likely to play a key role in preserving enzymatic structure and activity. Thr_245_, located within α-helix 6, is close to the main binding-specificity pockets, next to the pepsin cleavage site (Arg_238_-Arg_239_), which enhances the catalytic efficiency of CPN.^42^ A Thr-to-Met transition is predicted to destabilize the Pro_225_ position, and consequently the nearby loop—Asn_223_ and Asp_227_—lining the pocket recognizing the Arg/Lys to be cleaved. The role of Gly_178_ has not yet been fully deciphered; a Gly-to-Asp transition may disrupt the conformation of the Gly_177_-Gly_178_ stretch that stabilizes the structure after α-helices 4/5. On the basis of the predicted 3-dimensional structure published,^42^ missense variants at both these positions could affect binding of the regulatory CPN2 subunit. The carbohydrates are also displayed on the same face of the enzyme and could possibly modulate catalytic activity of the CPN1 subunit.

Additional *CPN1* variants were recently reported in HAE families in combination with other alleles, for example, a NM_001308.2:c.931T>C;p.(Cys311Arg) variant, predicted to be deleterious, combined with NM_000301.3(PLG):c.988A>G;p.(Glu330Lys) in a patient with HAE with plasminogen activation,^43^ and a NM_001308.2:c.1219G>A;p.(Glu407Lys) variant combined with *TLR4*, *MASP1*, *PLAU*, and *MPO* variants in a patient with HAE of unknown cause.^26^ Neither report documented biological data for CPN activity.

Low CPN activity has been implicated in protamine-reversal syndrome, a severe iatrogenic condition. Protamine, with its neutralizing properties of the effects of heparin, is given after extracorporeal circulation. But in patients with protamine-reversal syndrome, it can trigger a catastrophic reaction resulting in pulmonary vasoconstriction, bronchoconstriction, and systemic hypotension. Because protamine is a potent CPN inhibitor, a decreased anaphylatoxin and kinin inactivation has been suggested to contribute to the syndrome.^44^ More generally, CPN has been recognized as a pleiotropic regulator of inflammation.^21^^,^^45^^,^^46^ Furthermore, decreased plasma CPN activity has been identified as a risk factor for disease severity in patients with HAE-C1-INH^47^ and HAE-FXII.^48^

CPN, more than CPB2, is a potent C3a and C5a inactivator.^12^ The observation of urticaria in the CPN-deficient families described here is compatible with the anaphylatoxin properties described earlier.^10^ Plasma from *Cpn1*^−/−^ mice failed to cleave the C-terminal Arg from C3a and C5a, emphasizing an important role that CPN plays in anaphylatoxin inactivation.^46^ CPN has been demonstrated to protect from vascular leakage.^18^ These observations are congruent with a regulation by CPN of the biologically active anaphylatoxins and kinins. In addition to the vascular effects, anaphylatoxins are nonimmune activators of mast cell, with involvement in the pathophysiology of urticaria.

In addition, CPN may participate in plasminogen activation control. Through its catalytic action, CPN removes C-terminal Lys residues from cell surface proteins that act as plasminogen “receptors”; the binding of plasminogen to C-terminal Lys residues on cell surface enhances its activation up to 1000-fold.^49^ Consequently, CPN can downregulate plasminogen activation. Accordingly, it is tempting to speculate that when CPN activity is decreased in plasma, plasmin activity is likely to increase, leaving the KKS prone to rapid activation. This scenario is compatible with our observation of high-molecular-weight kininogen cleavage in plasma during acute symptoms in patients’ plasma with low CPN activity (not shown), leading to bradykinin production. The hypothesis could be consistent with the herein reported positive response of CPN-deficient patients to prophylaxis with tranexamic acid.

Mixed angioedema and urticaria phenotypes have already been recorded.^50^^,^^51^ The present observations are in agreement with this phenotype. However, the angioedema episodes described herein occurred in a reduced kinin catabolism condition, similarly to iatrogenic angioedema with ACEi and dipeptidyl-peptidase 4 inhibitors.^50^ Some hereditary situations were provisionally included in an informal group with HAE of unknown cause. On the basis of the results presented here, we suggest that CPN deficiency could characterize a group with HAE-CPN, with consequent challenges for patient treatment. Indeed, symptoms in these families do not respond to H_1_ antihistamines, and prophylaxis currently recommended for HAE must therefore be adapted.

Our work implicates CPN mutations and enzyme deficiency in contributing to angioedema symptoms in HAE-nC1-INH. CPN deficiency could impair bradykinin catabolism and thereby increase B_2_ receptor activation. As for other HAE-nC1-INH types, diagnosis of HAE-CPN requires concerted clinical, biological, and genetic investigation to decipher the dysregulation of the kallikrein-kinin pathway.

## Disclosure statement

This work was supported by an 10.13039/100017732E-Rare-1 research grant attributed within European FP7 (HAEIII; S. Cichon, coordinator) and a French National Agency for Research grant (grant no. EudraCT #38RC09.023). The promoter for the study was CHU Grenoble Alpes (#2009-A00025-52). Funding was also obtained from the French National Blood Service (Etablissement Français du Sang) La Plaine Saint Denis (grant no. APR2016-64), from KininX SAS, and the National Rare Disease Program from the French Ministry of Health (National Reference Center for Angioedema CREAK). F.P. was recipient of a PhD fellowship from Etablissement Français du Sang (#APR2016-64).

Disclosure of potential conflict of interest: F. Parsopoulou, G. Loules, and A. Ghannam received grants as stated in the funding section. The rest of the authors declare that they have no relevant conflicts of interest.
