# Corrigendum

**DOI:** 10.1016/j.jacig.2024.100319

**Published:** 2024-08-02

With regard to the article entitled ‘‘Hereditary angioedema with normal C1 inhibitor associated with carboxypeptidase N deficiency ’’ (J Allergy Clin Immunol Global 2024;3:100223), the authors have notified the Editors of a necessary change in Table I and Fig 1. In Table I, Family A should be corrected with both asymptomatic mother II.3 and daughter III.1. Additionally, in Fig 1 Family C should be corrected by introducing the synonymous variant c.1299T that is carried by both sister III.1 and mother II.2, and c.582G also carried by sister III.1. The Results section is correct. The authors apologize for the errors. The corrected Table I and Fig 1 appear below.

**Figure:**
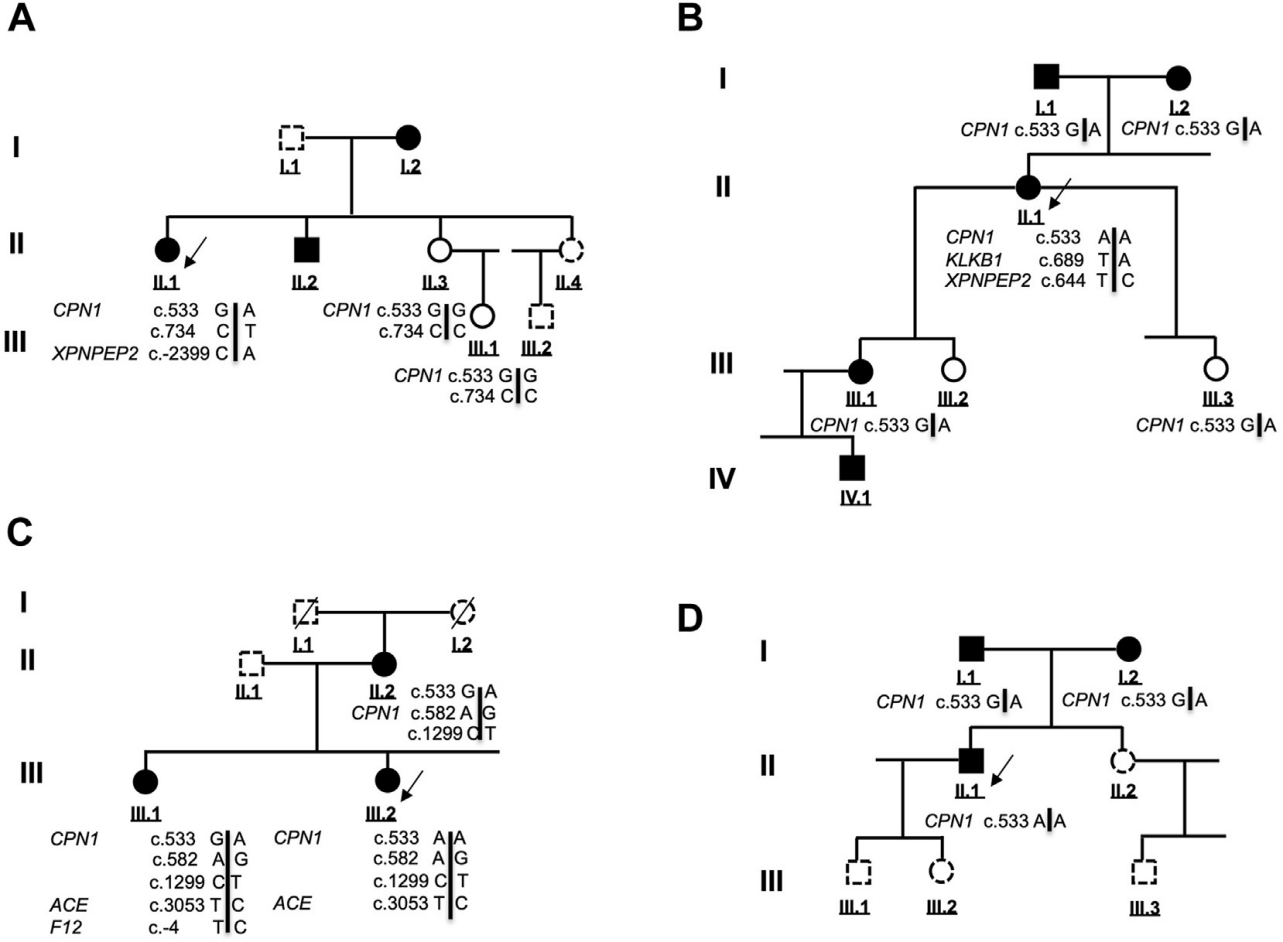

**Table I:** Clinical records

Family ID	Patient	Sex	Symptoms∗					Trigger	Age of onset	Delay diagnostic	Treatment prophylaxis
			Peripheral	Abdominal	Laryngeal	Macroglossia	Urticaria				
A	I.2	F	Yes	Yes	No	No	No	Unknown	40 y	1 y	Tranexamic acid
	II.1†	F	Yes	Yes	Yes	No	Yes	Pressure pruritus, triptorelin	41 y	2 y	Tranexamic acid, icatibant on demand
	II.2	M	Yes	Yes	Yes	No	Yes	Unknown	—	—	Tranexamic acid
	II.3	F	No	No	No	No	No	Unknown	—	—	None
	III.1	F	No	No	No	No	No	Unknown	—	—	None
B	I.1	M	No	No	No	No	Yes	Unknown	22 y	75 y	None
	I.2	F	Yes	Yes	No	No	Yes	None	27 y	70 y	None
	II.1†	F	Yes	Yes	Mild	No	Yes	Spontaneous and/or cold	30 y	12 y	Tranexamic acid, montelukast, icatibant on demand
	III.1	F	Yes	Yes	No	No	Yes	Unknown	25 y	1 y	Tranexamic acid, montelukast, icatibant on demand
	III.3	F	No	No	No	No	Yes	Unknown	17 y	1 y	None
	IV.1	M	No	Yes	No	No	Yes	Unknown	12 y	6 mo	None
C	II.2	F	Yes	Yes	No	No	Yes	Cold	47 y	6 mo	None
	III.1	F	No	Yes	No	No	Rare	Unknown	17 y	6 mo	None
	III.2†	F	—	Yes	No	No	Yes	Cold	15 y	6 mo	Tranexamic acid, icatibant on demand
D	I.1	M	No	Yes	No	No	Yes	Unknown	16 y	30 y	None
	I.2	F	No	No	No	No	Yes	Unknown	12 y	30 y	None
	II.1†	M	Yes	Yes	Yes	Rare	Chronic urticaria	Pressure, cold, fatigue	18 y	14 y	Tranexamic acid, montelukast, icatibant or C1-INH concentrate on demand

*F*, Female; *M*, male.

∗ Urticarial lesions in CPN-deficient patients developed frequently, but not consistently, in association with angioedema attacks. An urticarial rash accompanied nearly 60% of symptomatic episodes of angioedema.

† Family proband.

