# Supplementary material for: Hereditary angioedema with normal C1 inhibitor associated with carboxypeptidase N deficiency
Source: J Allergy Clin Immunol Glob. 2024 Feb 1;3(2):100223. doi: 10.1016/j.jacig.2024.100223 (PMC10912455; doi:10.1016/j.jacig.2024.100223)
Supplement: Table S1 [file mmc1.docx]

**Table S1**. Genes included in the by next-generation sequencing analysis.

| **Gene** | **Reference** | **Coverage %** | **Exon** | **Gene** | **Reference** | **Coverage %** | **Exon** |
| --- | --- | --- | --- | --- | --- | --- | --- |
| *A2M* | NM_000014.4 | 99.7 | 36 | *MASP1* | NM_139125.3 | 100 | 18 |
| *ACE* | NM_000789.3 | 97.4 | 26 | *MASP2* | NM_006610.3 | 94.8 | 12 |
| *AR* | NM_000044.3 | 92.3 | 9 | *MME* | NM_007288.2 | 100 | 22 |
| *BDKRB1* | NM_000710.3 | 100 | 1 | *MPO* | NM_000250.1 | 97.4 | 12 |
| *BDKRB2* | NM_000623.3 | 100 | 2 | *PLAT* | NM_000930.3 | 100 | 13 |
| *C1QBP* | NM_001212.3 | 100 | 6 | *PLAU* | NM_001001791.2 | 100 | 11 |
| *C1R* | NM_001733.4 | 100 | 9 | *PLAUR* | NM_002659.3 | 93.8 | 8 |
| *C1S* | NM_201442.2 | 100 | 11 | *PLG* | NM_000301.3 | 95.9 | 20 |
| *CPM* | NM_001005502.2 | 100 | 8 | *PRCP* | NM_199418.2 | 100 | 10 |
| *CPN1* | NM_001308.2 | 92.3 | 9 | *PROC* | NM_000312.3 | 97.1 | 8 |
| *DPP4* | NM_001935.3 | 100 | 26 | *SERPINA1* | NM_001127700.1 | 100 | 4 |
| *ELANE* | NM_001972.2 | 89.7 | 5 | *SERPINB2* | NM_002575.2 | 100 | 7 |
| *ESRRA* | NM_004451.4 | 94.0 | 7 | *SERPINC1* | NM_000488.3 | 100 | 7 |
| *F11* | NM_000128.3 | 100 | 14 | *SERPINE1* | NM_000602.4 | 100 | 8 |
| *F12* | ΝΜ_000505.3 | 96.8 | 14 | *SERPINF1* | NM_002615.5 | 97.0 | 7 |
| *F13A1* | NM_000129.3 | 100 | 14 | *SERPING1* | ΝΜ_000062.2 | 100 | 8 |
| *F13B* | NM_001994.2 | 100 | 12 | *TAC1* | NM_003182.2 | 100 | 6 |
| *F2* | NM_000506.3 | 95.7 | 14 | *TLR2* | NM_003264.3 | 100 | 1 |
| *GPER1* | NM_001098201.1 | 100 | 1 | *TLR4* | NM_138554.4 | 100 | 5 |
| *HRH1* | NM_001098212.1 | 100 | 1 | *TLR9* | NM_017442.3 | 97.7 | 2 |
| *HSP90AA1* | NM_001017963.2 | 99.5 | 12 | *TNF* | NM_000594.3 | 100 | 4 |
| *IL5* | NM_000879.2 | 100 | 4 | *TPSAB1* | NM_003294.3 | 66.9 | 5 |
| *KLK1* | NM_002257.3 | 100 | 5 | *TPSD1* | NM_012217.2 | 98.1 | 5 |
| *KLK2* | NM_005551.4 | 100 | 8 | *TPSG1* | NM_012467.3 | 100 | 6 |
| *KLK3* | NM_001648.2 | 98.9 | 7 | *XPNPEP1* | NM_020383.3 | 100 | 21 |
| *KLKB1* | NM_000892.3 | 100 | 14 | *XPNPEP2* | NM_003399.5 | 91.2 | 21 |
| *KNG1* | NM_001102416.2 | 99.2 | 12 | *XPNPEP3* | NM_022098.3 | 98.6 | 11 |
| *KRT1* | NM_006121.3 | 100 | 9 |  |  |  |  |
